# Intensity-modulated radiation therapy with simultaneous integrated boost for locally advanced breast cancer: a prospective study on toxicity and quality of life

**DOI:** 10.1038/s41598-019-39469-8

**Published:** 2019-02-26

**Authors:** David Pasquier, Florence Le Tinier, Raoudha Bennadji, Anais Jouin, Samy Horn, Alexandre Escande, Emmanuelle Tresch, Marie Pierre Chauvet, Audrey Mailliez, Frederik Crop, Xavier Mirabel, Eric Lartigau

**Affiliations:** 1 Academic Department of Radiation Oncology, Centre Oscar Lambret, 3 rue Frédéric Combemale, F-59000 Lille, France; 2 CRIStAL UMR CNRS 9189, Université Lille, Avenue Carl Gauss, F-59650 Villeneuve-d’Ascq, France; 3 Methodology and Biostastistics Unit, Centre Oscar Lambret, 3 rue Frédéric Combemale, F-59000 Lille, France; 4 Department of Surgery, Centre Oscar Lambret, 3 rue Frédéric Combemale, F-59000 Lille, France; 5 Department of Medical Oncology, Centre Oscar Lambret, 3 rue Frédéric Combemale, F-59000 Lille, France; 6 Department of Medical Physics, Centre Oscar Lambret, 3 rue Frédéric Combemale, F-59000 Lille, France

## Abstract

Radiotherapy after breast conserving surgery and mastectomy with node positive disease has been shown to reduce risk of recurrence and mortality in the treatment of breast cancer. Intensity-modulated radiation therapy (IMRT) after conservative surgery offers several advantages over conventional RT including improved acute and late toxicity and quality of life (QoL). We undertook this study to prospectively evaluate acute (≤90 days after last dose of radiotherapy) and long-term (>90 days) cutaneous, esophageal, and fibrosis toxicity and QoL in breast cancer patients treated by adjuvant IMRT after breast surgery. We included patients with complex volumes for which 3D RT does not allow a good coverage of target volumes and sparing organs at risk. We report here an interim analysis with a median follow-up of 13.1 months (range, 6.5–25.9 months). Most of the acute toxicity was cutaneous (95.9%) and oesophageal (59.6%), and mostly grade 1 and 2. Medium-term cutaneous toxicity rate was 25.6%, and mostly grade 1. Medium-term esophageal toxicity was rare (1.8%). In this series acute oesophageal toxicity was found to be associated with dosimetric factors. QoL was well preserved throughout the study, and aesthetic outcomes were good. Based on these data, tomotherapy may be a favorable alternative to other techniques in patients needing a complex irradiation of the breast and lymph node volumes.

## Introduction

Radiotherapy is recommended after breast conserving surgery and mastectomy with node positive disease in the treatment of breast cancer. A meta-analysis of data from over 10,000 women revealed that adjuvant radiotherapy reduces risk of recurrence by up to 15% and 15-year mortality rates by 4% in the treatment of breast cancer after breast-conserving surgery^1^. After total mastectomy, in the event of local lymph node invasion, radiotherapy allows reducing recurrence in up to 10% and the 20-year mortality rate in up to 8% of the cases, regardless of the number of invaded nodes^1^.

Adjuvant radiotherapy in the treatment of breast cancer, however, can result in acute and late adverse events. Acute toxicity affects notably the skin and esophagus, while late toxicity includes cosmetic and functional sequelae, lung fibrosis, cardiovascular toxicity and secondary cancers^2–6^.

3D conformational radiotherapy (3D-CRT) is the standard treatment but it presents some disadvantages including dose inhomogeneity leading to increased acute reactions, inadequate cosmetic outcomes^7^, organs at risk (OAR) toxicity and local recurrence risk^8^, as well as complex treatment settings in cases of associated lymph node irradiation.

Three recent randomized trials have demonstrated that intensity-modulated radiation therapy (IMRT) after conservative surgery offers several advantages over conventional RT including improved acute and late breast toxicity and quality of life (QoL)^9–13^. These trials, however, suffered from some limitations: included patients had an early stage breast cancer; treatment in the control arm was 2D-CRT, which is no longer the therapeutic standard; intervention in the experimental arm constituted a simplified IMRT technique; and few data were reported on local control and none on survival or late toxicity other than aesthetics.

Compared to 3D-CRT, IMRT allows covering target volumes while sparing OAR when radiating complex shapes. Even though the use of IMRT is increasing, a lack of available clinical data, notably regarding rotational IMRT, precludes its use in routine care^14^. Furthermore, IMRT increases the volume receiving low-dose with uncertainty regarding its long-term implications

The aim of this study was to evaluate acute and medium-term breast, cutaneous and esophageal toxicity in breast cancer patients treated by adjuvant IMRT. Secondary objectives were to analyze the association between clinical and dosimetric characteristics and toxicity, to evaluate QoL and aesthetic outcomes, and to compare the toxicity of the different delineation protocols used.

## Methods

This was a single-centre, prospective evaluation of the tolerance of adjuvant IMRT by tomotherapy in the treatment of breast cancer patients after breast surgery within routine care (ClinicalTrials.gov Identifier: NCT02281149). The study was approved by the local ethics committee (“Comité de Protection des Personnes Nord Ouest IV”) and conducted in accordance with the Helsinki declaration and good clinical practice guidelines. Informed consent was obtained from all patients. Inclusion criteria were patients ≥18 years, with histologically proven breast cancer, undergoing adjuvant radiotherapy after partial or total mastectomy, with or without irradiation of axillary lymph nodes. Patients with metastatic disease, presenting a severe or non-controlled pathology that could compromise participation in this trial, breast-feeding or pregnant, and unable to undergo medical follow-up for geographical, social or psychological reasons were excluded.

### Treatment procedure

Target and OAR volume delineation were performed according to ASTRO guidelines^15^ until January 2016 and ESTRO guidelines^16^ thereafter. A 5-mm margin was added to the clinical target volume (CTV) to obtain the planning target volume (PTV).

Prescribed dose to the breast/chest wall/axillary lymph nodes was 50 Gy (25 fractions x 2 Gy) to the breast over five weeks (5 irradiations/week) with simultaneous integrated boost (SIB) at surgical bed (PTV-boost) of 60 Gy in 25 fractions (25 fractions x 2.4 Gy) after conservative treatment. The goal of the prescription was that 95% of the volume received 95% of the prescribed dose.

The treatment procedure has been described previously^17,18^.

### Endpoints

The primary endpoint of the study was acute (≤90 days after last dose of radiotherapy) and long-term (>90 days) toxicity related to radiotherapy, determined according to NCI-CTCAE v4.0 criteria. Studied toxicities included cutaneous toxicity (described by the terms radiodermatitis, ulceration-necrosis, telangiectasia, atrophy, hyperpigmentation and hypopigmentation), esophageal toxicity, and breast fibrosis. Toxicities were assessed at baseline, during RT (one/week), at 1 and 6 months, and at 1, 2, 3, 4, 5 years post-radiotherapy. Acute toxicity was reported during radiotherapy and at 1-month follow-up. Medium-term toxicity was reported at 6-month follow-up or after. Secondary endpoints were QoL (evaluated by the EORTC QLQ-C30 and BR-23 questionnaires), aesthetic outcomes (coded as poor, medium, good, excellent and collected from the physician and the patient) and recurrence-free survival (RFS).

### Instruments for QoL

The EORTC QLQ-C30, is a cancer-specific measure of HRQOL. EORTC QLQ-C30 and QLQ-BR23 scorings were performed according to the EORTC manual^19^. Scores were linearly transformed to a 0-to-100 scale. A high or healthy level of functioning was represented by a high functional score. A high QoL is represented by a high score for global health status or QoL. More severe symptoms are expressed by higher scores in symptoms scales.

### Statistical considerations

Statistical analyses were performed using Stata 13.1 (StataCorp. 2013, College Station, Texas, USA).

Patient characteristics are presented using descriptive variables: frequencies and percentages for categorical variables; medians and ranges, and means and standard deviations (sd) for continuous variables. Cutaneous toxicity and fibrosis were measured per treated breast to include bilateral cancers. Esophageal toxicity was analyzed per patient. Acute toxicity was estimated as frequency and percentages by grade and toxicity type. Cumulative incidence of medium-term toxicity was estimated by the Kaplan-Meier method considering the time-lapse between the end of RT and the occurrence of late toxicity. Patients not presenting any toxicity were censored at the date of last news.

An analysis of the association between clinical and dosimetric characteristics and acute or medium-term cutaneous or esophageal toxicities was performed. Clinical parameters tested for cutaneous toxicity were age, BMI, breast volume, smoking status, diabetes, esthetical results prior to radiotherapy and prior chemotherapy. Dosimetric parameters tested for cutaneous toxicity were mean dose received (Dmean) and dose (Gy) received by 2, 50, 95, 98% of the volume (D2%, D50%, D95%, D98%) for the target volumes (breast, susclavian and subclavian), skin, susclavian and subclavian skin areas; volume (cc) of the target and skin areas, volume (cc) of the breast target volume receiving 95% of the dose (V95%). Clinical parameters tested for esophageal toxicities were age, BMI, smoking status, diabetes and prior chemotherapy. Dosimetric parameters tested for toxicity to the esophagus were volume of the esophagus, Dmean, D2%, D50%, D95%, D98%, volume (cc) of the esophagus that received a dose of 30 or 45 Gray (V30 Gy, V45 Gy). The analysis was performed on the overall population and on the total and partial mastectomy subgroups for cutaneous toxicities.

The association between acute toxicity considered as a binary variable with clinical and dosimetric characteristics was analyzed for qualitative variables with the chi-2 test or the Fisher exact test in case of a small sample. For quantitative variables we used the Student’s t-test if normality of variables or sample ≥30 or the Wilcoxon Mann-Whitney test otherwise. A multivariate analysis was then performed using logistical regression model.

The association between medium-term toxicity, considered as a time-to-event variable, with clinical and dosimetric characteristics was analyzed for qualitative variables with a Logrank test and for quantitative variables with a Cox univariate model. A multivariate analysis was performed using a multivariate Cox model.

For the selection of parameters to include in multivariate logistical and Cox regression models, variables significantly associated to toxicity (p < 0.05), or not significantly associated but with a p < 0.10 in univariate analysis were taken into account. In the case of highly correlated parameters (r² > 0.70), only the one associated to the endpoint with a better Akaike’s information criteria (lower AIC) was included in the multivariate model to avoid collinearity. A multivariate backward stepwise selection procedure was performed to keep only variables associated to toxicity with p < 0.05 and to remove variables insufficiently associated to the endpoint from the final multivariate model.

An exploratory analysis was performed to evaluate the association between acute esophageal toxicity and the whole range of dose received by percentage of the esophageal volume (from D1% to D100% (Gy)) using the Wilcoxon Mann-Whitney test.

RFS was estimated using the Kaplan-Meier method and was defined as the time between inclusion and the first sign of disease recurrence or death. Patients alive and recurrence-free at last follow-up were censored at the date of last news.

Scores corresponding to QLQ-C30 and BR23 were described with median, ranges, mean and sd values; score differences between different time-points and inclusion were also calculated. Score changes over time were evaluated with variance analyses variances for repeated measures.

## Results

One hundred and nineteen patients were included in Centre Oscar Lambret (Lille, France) between November 2014 and April 2016. Five patients left the study prematurely (n = 2 patient decision, n = 1 decision of the investigator, n = 1 change of RT technique, n = 1 disease progression). Baseline patient and tumor characteristics are presented in Table 1.

**Table 1:** Patients’ demographic and medical characteristics.

Characteristics	n	%
**Patients characteristics (n = 114)**		
Age (years), median (range)	56.0 (32.0–83.0)	
WHO (n = 109)		
0	91	83.5
1	18	16.5
BMI (n = 112)		
Median (range)	26.5 (16.5–48.8)	
Normal <25	42	37.5
Overweight 25–30	39	34.8
Obese ≥30	31	27.7
Smoking history	32	28.1
Pack per year (n = 29) Median (range)	15.0 (1.0–51.0)	
Duration (years) (n = 26) median (range)	27.0 (2.0–46.0)	
Lung history (n = 113)	9	8.0
Cardiovascular history (HTA, coronaropathy, heart failure) (n = 113)	35	31.0
Hypertriglyceridemia (n = 113)	6	5.3
Diabetes (n = 112)	11	9.8
Insulin dependent diabetes (n = 113)	3	2.7
Hypercholesterolemia (n = 113)	22	19.5
**Tumor characteristics** ^*^ **(n = 121)**		
Side of tumor^$^		
Right	59	48.8
Left	62	51.2
Histology		
Invasive ductal carcinoma	92	76.0
Invasive lobular carcinoma	15	12.4
Other	14	11.6
*In situ* component	46	38.0
SBR		
Non-gradable (after neo adjuvant chemotherapy)	20	16.5
SBR I	26	21.5
SBR II	57	47.1
SBR III	18	14.9
ER+ (n = 117)	105	89.7
PR+ (n = 117)	85	72.6
HER2+ (n = 95)	12	12.6
Triple negative	2	1.7
pT (n = 107)		
pT1	48	44.9
pT2	42	39.3
pT3	16	15.0
pT4	1	0.9
pN (n = 111)		
pN0	20	18.0
pN1	62	55.9
pN2	23	20.7
pN3	6	5.4

^*^Data are described by treated breast.

^$^9 patients presented with bilateral BC: seven patients were treated by RT in both sides; 2 patients were treated on a single side.

Abbreviations: WHO = world health organization; BMI = body mass index; BC = breast cancer; ER = estrogen receptor; PR = progesterone receptor; HR = hormonal receptor; SBR = Scarff-Bloom et Richardson histopronostical grade; pT = anatomopathological TNM classification of primary tumor; pN = anatomopathological TNM classification of regional nodes

The median dose prescribed to the breast/chest wall/axillary nodes PTV was 50 Gy (range, 40.0–50.5 Gy) and a median simultaneous boost to the surgical bed of 10 Gy (range, 9.6–15.0 Gy). Table 2 presents the treatment administered to patients.

**Table 2:** Treatment received.

Treatment type	N	%
Surgery* (N = **121)**		
Partial mastectomy	63	52.1
Total mastectomy	58	47.9
Sentinel lymph node	70	57.9
Axillary node dissection	101	83.5
**Chemotherapy**^$^ **(N** = **114)**		
Adjuvant	61	53.5
Neo-adjuvant	29	25.4
Any/at any time	88	77.2
**Hormone therapy**^**$**^ **(N** = **114)**		
Any drug	93	81.6
Tamoxifen-based	36	31.6
Aromatase inhibitor-based	49	43.0
Other	8	7.0
Radiotherapy^*^(N = **121)**		
Breast or chest wall PTV	121	100.0
D50% (mean ± SD) (Gy)	49.7 ± 1.3	
D95% (mean ± SD) (Gy)	46.2 ± 4.3	
D2% (mean ± SD) (Gy)	56.3 ± 4.4	
Concomitant Boost PTV	67	55.4
D50% (mean ± SD) (Gy)	59.4 ± 1.6	
D95% (mean ± SD) (Gy)	56.9 ± 1.8	
D2% (mean ± SD) (Gy)	61.5 ± 1.6	
Internal mammary chain PTV	113	93.4
D50% (mean ± SD) (Gy)	49.4 ± 2.6	
D95% (mean ± SD) (Gy)	46.6 ± 5.3	
D2% (mean ± SD) (Gy)	52.1 ± 2	
Subclavicular PTV	110	90.9
D50% (mean ± SD) (Gy)	49.5 ± 2.2	
D95% (mean ± SD) (Gy)	46.8 ± 3.1	
D2% (mean ± SD) (Gy)	51.9 ± 1.6	
Supraclavicular PTV	110	90.9
D50% (mean ± SD) (Gy)	49.4 ± 2.1	
D95% (mean ± SD) (Gy)	46.6 ± 3.3	
D2% (mean ± SD) (Gy)	52 ± 1.5	

^*^By treated breast.

^$^By patient.

PTV: planning target volume.

Dx %: dose received by at least x% of the volume.

Median treatment time was 36 days (range, 21–44 days), and median follow-up 13.1 months (range, 6.5–25.9 months) since the start of RT and 11.9 months (range 5.4–24.7 months) since the end of RT.

Table 3 presents toxicity incidences by maximum grade. Most common acute skin toxicities were radiodermitis (109/121, 90.1%) and hyperpigmentation (82/121, 67.8%), which were mostly grade 1 (80/121, 66.1%). There were two grade 3 acute radiodermitis. Most common medium-term toxicities were hyperpigmentation (9/121, 7.4%), atrophia (8/121, 6.6%) and telangiectasis (8/121, 6.6%). The only case of medium-term grade 3 toxicity was a ulceration-necrosis. The maximum acute esophageal toxicity was grade 1 in 59/114 (51.8%) patients and grade 2 in 9/114 (7.9%) patients. Medium-term esophageal toxicity was rare with 2/114 (1.8%) patients experiencing grade 1 events. Forty-six of 114 (38.0%) patients experienced acute fibrosis, none grade 2. Medium-term fibrosis of grade 2 was reported by 8/114 (6.6%) patients.

**Table 3:** Toxicity profile (by maximum grade).

Toxicity	Acute		Medium-term	
	N	%	N	%
**Skin**^**a**^ (**N** = **121)**				
Grade 1	74	61.2	28	23.1
Grade 2	40	33.1	1	0.8
Grade 3	2	1.7	1	0.8
Unknown	—	—	1	0.8
All grades	116	95.9	31	25.6
**Fibrosis**^**a**^ **(N** = **121)**				
Grade 1	N/A	N/A	27	22.3
Grade 2	N/A	N/A	8	6.6
Unknown	N/A	N/A	1	0.8
All grades	N/A	N/A	36	29.8
**Esophageal** ^**b**^ **(N = 114)**				
Grade 1	59	51.8	2	1.8
Grade 2	9	7.9	0	0
All grades	68	59.6	2	1.8

Footnotes:

^a^Per breast.

^b^Per patient.

*Skin toxicity is defined by the following terms: radiodermatitis, ulceration-necrosis, telangiectasia, atrophy, hyperpigmentation, hypopigmentation.

**Late skin toxicity and fibrosis: we only considered events that had not been reported as acute toxicities or whose grade increased.

Abbreviations: N/A = non-applicable.

One-year cumulative incidences of medium-term grade ≥2 skin and esophageal toxicity, and grade ≥2 fibrosis were 4.1% (95% CI, 1.1–15.6), 8.7% (95% CI, 4.3–17.3) and 0%, respectively.

Multivariate analysis revealed a significant association between skin grade ≥2 acute toxicity and BMI (p = 0.003; OR = 1.14, 95% CI, [1.04–1.24]), dose received by 95% of the skin volume (D95%) (p = 0.033; OR = 1.11, 95% CI, [1.01–1.22]) and susclavian skin D98% (p = 0.034; OR = 1.04, 95% CI [1.003–1.09]) (Table 4). In the total mastectomy sub-group analysis, a higher D98% susclavian skin value was associated with a higher risk of developing skin grade ≥2 acute toxicity (p = 0.013; OR = 1.07, 95% CI [1.016–1.135]). In the partial mastectomy group, skin grade ≥2 acute toxicity was significantly associated with BMI (p = 0.015; OR = 1.17, 95% CI [1.03–1.32]) and chemotherapy (p = 0.001; OR = 0.09, 95% CI [0.02–0.39]). Not enough events were recorded to perform association analyses with skin grade ≥2 medium-term toxicities. Only volume of subclavian skin was associated with skin grade ≥1 medium-term toxicity (p = 0.049; HR = 1.02, 95% CI [1.00–1.05]) (Table 5). In the total and partial mastectomy subgroups, not enough events occurred to perform multivariate analyses.

**Table 4:** Association between acute grade ≥2 cutaneous toxicity and clinical and dosimetric characteristics

	N	Acute grade ≥2 cutaneous toxicity		Univariate analysis*	Multivariate analysis**	
		n	%	p-value	OR (95%CI)	p-value
**Age (years)**	—	—	—	0.51	NA	
**BMI (kg/m²)**	—	—	—	0.003	1.14 (1.05–1.24)	0.003
**Bra cup size (n = 113)**				0.020	ND	
A-B	53	12	22.6%			
≥C	60	26	43.3%			
**Smoking**				0.44	NA	
No	86	28	32.6%			
Yes	35	14	40.0%			
**Diabetes (n = 119)**				0.52	NA	
No	108	37	34.3%			
Yes	11	5	45.5%			
**Chemotherapy**				0.017	0.49 (0.16–1.49)***	0.21
No	28	15	53.6%			
Yes	93	27	29.0%			
**Aesthetic aspect before RT (n = 66)**				0.70	NA	
Moderate	14	6	42.9%			
Good	33	10	30.3%			
Excellent	19	7	36.8%			
**Dosimetric parameters**						
Volume of breast CTV (cc)	—	—	—	0.064	ND	
Volume of breast PTV (cc)	—	—	—	0.055	ND	
Volume of subclavian CTV (cc)	—	—	—	0.064	0.98 (0.969–1.001)***	0.051
Volume of subclavian PTV (cc)	—	—	—	0.070	ND	
Dmean (Gy) for the skin (Gy)	—	—	—	0.045	ND	
D50% for the skin (Gy)	—	—	—	0.014	ND	
D95% for the skin (Gy)	—	—	—	0.047	1.11 (1.01–1.22)	0.033
D95% for the susclavian skin (Gy)	—	—	—	0.089	ND	
D98% for the susclavian skin (Gy)	—	—	—	0.047	1.04 (1.003–1.09)	0.034
V95% of CTV breast (cc)	—	—	—	0.068	1.00 (0.992–1.014)***	0.56
V95% of PTV breast (cc)	—	—	—	0.072	ND	

CTV: clinical target volume; PTV: planning target volume; RT: radiotherapy; OR: odds ratio; CI: confidence interval.

Dx%: dose (Gy) received by x% of the volume; Vx%: volume (cc) of the breast target volume that received x% of the dose.

*Univariate analysis: Khi-2 test, Student t-test or Wilcoxon test.

**Multivariate analysis: logistic regression.

NA: not applicable because not included in multivariate regression model (p > 0.10 in univariate analysis).

ND (not done): variable not included in multivariate analysis for collinearity reasons (bra cup size, volume of breast CTV and PTV: highly correlated to BMI; subclavian PTV volume: highly correlated to subclavian CTV volume; Dmean and D50% to skin: highly correlated to D95% to skin; D95% to susclavian skin: highly correlated to D98% to susclavian skin; V95% of PTV: highly correlated to V95% of CTV).

***Variable included in multivariate analysis but removed from the model because of the stepwise procedure.

**Table 5:** Association between medium-term grade ≥1 cutaneous toxicity and clinical and dosimetric characteristics.

		Medium-term grade ≥1 cutaneous toxicity		Univariate analysis*	Multivariate analysis**	
	N	n	%	p-value	HR (95%CI)	p-value
**Age (years)**	—	—	—	0.51	NA	
**BMI (kg/m²)**	—	—	—	0.43	NA	
**Bra cup size (n = 113)**				0.67	NA	
A-B	53	12	22.6%			
≥C	60	16	26.7%			
**Smoking**				0.81	NA	
No	86	21	24.4%			
Yes	35	10	28.6%			
**Diabetes (n = 119)**				0.20	NA	
No	108	30	27.8%			
Yes	11	1	9.1%			
**Chemotherapy**				0.11	NA	
No	28	4	14.3%			
Yes	93	27	29.0%			
**Aesthetic aspect before RT (n = 66)**				0.36	NA	
Moderate	14	1	7.1%			
Good	33	9	27.3%			
Excellent	19	5	26.3%			
**Dosimetric parameters**						
Volume of subclavian skin (cc)	—	—	—	0.052	1.02 (1.001–1.05)	0.049
D2% for subclavian PTV (Gy)	—	—	—	0.084	1.13 (0.67–1.89)***	0.65
D2% for susclavian PTV (Gy)	—	—	—	0.019	0.78 (0.58–1.04)***	0.10
D50% for breast CTV (Gy)	—	—	—	0.047	0.96 (0.64–1.46)***	0.86

CTV: clinical target volume; PTV: planning target volume; RT: radiotherapy.

Dx%: dose (Gy) received by x% of the volume; Vx%: volume (cc) of the breast target volume that received x% of the dose.

HR: hazard ratio; CI: confidence interval.

*Univariate analysis: Logrank test or univariate Cox model.

**Multivariate analysis: Cox model.

NA: not applicable because not included in multivariate regression model (p > 0.10 in univariate analysis).

***Variable included in multivariate analysis but removed from the model because of the stepwise procedure.

No factor among those listed in Table 6 was significantly associated to acute grade ≥2 esophageal toxicity in univariate analysis. The percentage of esophagus volume receiving ≥30 Gy (V30) was at the limit of significance (p = 0.055). In univariate analysis, acute grade ≥1 esophageal toxicity was significantly associated to esophageal D2% (p = 0.0004; mean D2% = 43.1 Gy [sd 5.9] in patients with toxicity vs 37.2 Gy [sd 9.3] in patients without toxicity), esophageal mean dose (Dmean) (p = 0.009; mean Dmean = 14.5 Gy [sd 3.2] vs 13.1 Gy [sd 3.0]), esophageal V30 (p = 0.016; mean V30 Gy = 16.5% [sd 8.8] vs 11.1% [sd 9.1]) and esophageal V45 (p = 0.006; mean V45 Gy = 3.0% [sd 3.7] vs 1.5% [sd 2.8]), with a higher dose and a higher volume associated with a higher risk of grade ≥1 toxicity. These parameters were too highly correlated with each other to be included in a multivariate model.

**Table 6:** Association between acute grade ≥2 and grade ≥1 esophageal toxicity and clinical and dosimetric characteristics.

		Acute grade ≥2 cutaneous toxicity		Univariate analysis*	Acute grade ≥1 cutaneous toxicity		Univariate analysis*
	N	n	%	p-value	n	%	p-value
**Age (years)**	—	—	—	0.62	—	—	0.67
**BMI (kg/m²)**	—	—	—	0.82	—	—	0.55
**Smoking**				0.11			0.97
No	82	4	4.9%		49	59.8%	
Yes	32	5	15.6%		19	59.4%	
Diabetes (n = 112)				0.60			0.13
No	101	9	8.9%		59	58.4%	
Yes	11	0	0.0%		9	81.8%	
**Chemotherapy**				0.68			0.50
No	26	1	3.8%		17	65.4%	
Yes	88	8	9.1%		51	58.0%	
**Dosimetric parameters**							
Volume of the esophagus (cc)	—	—	—	0.53	—	—	0.78
Dmean to esophagus (Gy)	—	—	—	0.084	—	—	0.009
D2% to esophagus (Gy)	—	—	—	0.14	—	—	0.0004
D50% to esophagus (Gy)	—	—	—	0.84	—	—	0.80
D95% to esophagus (Gy)	—	—	—	0.44	—	—	0.45
D98% to esophagus (Gy)	—	—	—	0.91	—	—	0.57
V30 Gy to esophagus (cc)	—	—	—	0.055	—	—	0.0016
V45 Gy to esophagus (cc)	—	—	—	0.084	—	—	0.0006

Dx%: dose (Gy) received by x% of the esophageal volume; VxGy: volume (cc) of the esophagus that received a dose of x Gray.

*Univariate analysis: Khi-2 test, Student t-test or Wilcoxon test.

For grade ≥2 toxicity: there was not enough events to perform multivariate analysis.

For grade ≥1 toxicity: significant factors in univariate analysis (Dmean, D2%, V30 Gy, V45 Gy) are all too highly correlated (r² > 0.70) to be included in the same multivariate model.

The exploratory analysis for the association between acute esophageal toxicity and the dose received by percentage of the esophageal volume ranging from 1 to 100% (Gy) revealed a significant association between acute grade ≥2 esophageal toxicity and dose ranging from D7% to D36%, and between acute grade ≥1 esophageal toxicity and dose ranging from D1% to D30%.

QoL questionnaires were completed by 84.3% of patients at baseline, 86.8% at 1 month, 79.6% at 6 months and 73.9% at 1 year. Table 7 presents the scores at each time-point and the results for variance analyses for items related to local RT treatment.

**Table 7:** Quality of life of global population.

QLQ-C30 and BR-23	Scores Mean, (SD)				Score variation from inclusion Mean (SD)			
	Inclusion	1 month	6 months	1 year	1 month	6 months	1 year	p-value
**Global Health Status**	63.7 (18.5)	71.5 (17.9)	75.5 (17.5)	71.0 (19.4)	6.1 (18.5)	9.9 (18.7)	7.0 (17.9)	**<0.0001**
**FUNCTIONAL SCALES**								
Body image	57.4 (36.1)	66.7 (31.7)	72.8 (28.9)	75.8 (24.8)	7.8 (±22.4)	10.8 (24.5)	15.4 (25.7)	**<0.0001**
**SYMPTOMS**								
Breast symptoms	20.1 (18.4)	30.9 (20.1)	22.8 (17.6)	22.0 (17.4)	12.7 (19.0)	4.0 (21.9)	0.8 (19.7)	**<0.0001**
Arm symptoms	24.8 (22.1)	22.1 (21.1)	26.8 (19.8)	28.8 (24.9)	−0.4 (21.6)	5.7 (23.8)	1.9 (26.1)	0.06

Abbreviations: SD = standard deviation.

Global health status scores at 1-, 6- and 12-month follow-up were significantly higher than at inclusion (p < 0.0001), representing a higher level of QoL. Scores of global health status did not differ significantly during follow-up.

Body image scores improved significantly at 1 month, 6 months and 1 year compared to inclusion, reflecting a better body image. The score at 1 year was significantly higher than that at 1 month.

Score for breast symptoms at 1-month follow-up was significantly higher than that at inclusion, reflecting a worsening of symptoms. Scores at 6 months and 1 year were significantly lower than at 1 month, and did not differ significantly from those at inclusion.

At 1-year post RT, 21/37 patients (56.8%) experienced good aesthetic results, followed by 11/37 (29.7%) experiencing excellent results and 5/37 (13.5%) reporting moderate outcomes. Patient reported outcomes were in line with those reported by the physician (18/34 [52.9%] good outcomes, 10/34 [29.4%] excellent and 6/34 [17.6%] moderate).

The median follow-up (13.1 months) was too short to estimate late toxicity and RFS; only two recurrences and no death were reported.

## Discussion

Many have reported on the benefit of using a simultaneous boost to the tumor bed in reducing treatment time without compromising local control^20–24^. Tomotherapy is an improved radiation technique that allows for a better tumor coverage and sparing normal tissue in lungs and heart from high radiation doses compared to 3D-CRT.

Nevertheless, not many reports exist on the acute and late toxicity profiles of this technique in patients undergoing RT after lumpectomy or mastectomy. To our knowledge, this is one of the largest prospective series assessing the effect of a short strategy of 25 fractions with SIB in the tumor site on acute and medium-term toxicities, and QoL.

This study included patients with complex volumes for which 3D RT does not allow a good coverage of target volumes and sparing OARs. Most of the acute toxicity was cutaneous (95.9%) and oesophageal (59.6%), and mostly grade 1 and 2.

In most breast IMRT studies, lymph node areas and the esophagus were spared, and only a few have reported on oesophageal toxicity. Caudrelier *et al*. carried out a study of IMRT delivered by helical tomotherapy for locoregional breast radiation. Esophageal toxicity (dysphagia from esophagitis) was found in 37% of the 30 patients and was grade 1^25^. Aoulad *et al*. have recently reported the acute toxicity profile of intensity modulated helical tomotherapy during breast cancer irradiation after conserving surgery or mastectomy^26^. Acute grade 1–2 esophageal toxicities occurred in 19.9% of 292 patients. The lower rate in these studies could be attributed to the smaller sample size in the first one and the retrospective character in Aoulad’s study. Retrospective studies are known to minimize toxicity, particularly the low-grade toxicities that are most common in our study.

To our knowledge, this is the first series to describe a correlation between acute esophageal toxicity and dosimetric factors in this setting. In univariate analysis, acute grade ≥1 esophageal toxicity was significantly associated to esophageal D2% (p = 0.0004), Dmean (p = 0.009), V30 (p = 0.016) and V45 (p = 0.006). Only Aoulad *et al*. performed this analysis, but found no factor associated with acute esophageal toxicity^26^. In our study no variable was significantly associated with esophageal grade ≥2 toxicity. Esophageal V30 was at the limit of significance (p = 0.055). In light of these results, we are currently trying to minimize the dose to the esophagus.

Concerning acute skin toxicity, results are heterogeneous among studies. In our study, 2/3 of skin toxicities were of grade 1 and 1/3 of grade 2. Three studies reported slightly lower skin toxicity rates. In Franco *et al*.’s prospective study, 120 early breast cancer patients underwent whole breast IMRT after conserving surgery delivered with static angle tomotherapy^27^. Maximum detected acute skin toxicity was grade 0 for 22%, grade 1 for 63%, grade 2 for 12% and grade 3 for 3% of patients. Ha *et al*. retrospectively analyzed 214 patients with early stage breast cancer who were treated with breast conserving surgery followed by forward IMRT and boost to the surgical bed. Most patients had grade 0 or 1 acute toxicity (82.2%). Two patients reported grade 3 skin desquamation and no grade 4 acute toxicity was observed^28^. In Wojcieszynski *et al*.’s evaluation of two simultaneous integrated boost treatment planning techniques using helical tomotherapy for breast conserving therapy, 8/16 patients had grade 2 erythema immediately after irradiation^24^.

Skin toxicity has been shown to be associated with several patient- and treatment-related variables, such as BMI. In our series, median BMI was high, which could explain the higher incidence of acute toxicity. In this series, BMI (p = 0.003), skin D95% (p = 0.033) and susclavian skin D98% (p = 0.034) were associated with skin grade ≥2 acute toxicity. Despite the correlation with skin toxicity, optimizing the dose to the skin is difficult due to its thickness and the necessity of treating the target volume at the prescribed dose. In order to guarantee dose coverage and robustness of the treatment, dose to the skin should not be optimized^18^.

De Langhe *et al*. reported that bra cup size ≥ D, BMI, smoking during RT, and the use of concomitant hormone therapy were associated with acute grade ≥2 dermatitis in multivariate analysis^29^. Furthermore, patients treated with hypofractionated radiotherapy and those treated in prone position developed less dermatitis. In Franco *et al*.’s study no categorical variable was found to predict acute toxicity, while several continuous variables (volume of WB-PTV minus TB-PTV receiving 105, 110, 115% of prescription dose, whole breast and boost volume, breast thickness and soft tissue thickness) were associated with grade 2 and grade 3 skin acute events^27^.

Surprisingly, in univariate analysis, absence of chemotherapy was significantly associated with acute skin toxicity on the partial mastectomy subgroup (63 patients). Subclavian skin volume was associated with medium-term skin grade ≥1 toxicity in multivariate analysis, a variable that did not appear as a prognostic factor in other studies. In Florentino *et al*.’s study grade 2 acute skin toxicity was significantly associated with late grade 1 toxicity^30^.

Globally, QoL improved throughout the study. To date, few prospective or retrospective studies have reported QoL data in this context, rendering comparison difficult. In Franco *et al*.’s study, QoL was generally preserved^27^. Donovan and Pignol explored QoL outcomes in two randomized trials comparing IMRT and conventional RT for the treatment of breast cancer^9,10^. There was no difference in QoL between the experimental and the standard treatment arm.

In our series, most patients with positive axillary lymph nodes have been treated with chemotherapy. The results cannot therefore be generalized to all breast cancer patients benefiting from adjuvant RT and should be directed to those receiving IMRT. Additionally, the included population was heterogeneous concerning breast and parietal radiotherapy with or without axillary irradiation. Nevertheless, such heterogeneity brings this study closer to a real-life setting. Finally, sub-group analyses did not have enough power, and certain multivariate analyses were not performed.

Compared to 3D-CRT, IMRT provides excellent coverage of the target volume with lower volumes of OAR receiving high doses. IMRT delivers lower doses to larger volumes of the contralateral lung, contralateral breast, and other normal tissues, which could, in some cases, increased the risk of second cancer. Nevertheless, the risk-benefit ratio could be in favour of IMRT in complex target volumes [14]. This must be confirmed in large prospective series.

In conclusion, there were few statistically significant declines in QoL scores, and aesthetic outcomes were good. Based on these data, tomotherapy may be a favorable alternative to other techniques in patients needing a complex irradiation of the breast and lymph node volumes. Our study is ongoing and further studies will focus on longer follow-up in order to confirm these results.

## Data Availability

The datasets generated during and/or analyzed during the current study are not publicly available due to the clinical and confidential nature of the material but can be made available from the corresponding author on reasonable request.
